# Peroxidase-Like Platinum Clusters Synthesized by *Ganoderma lucidum* Polysaccharide for Sensitively Colorimetric Detection of Dopamine

**DOI:** 10.3390/molecules26092738

**Published:** 2021-05-06

**Authors:** Xiang Lai, Yu Han, Jie Zhang, Jinyu Zhang, Weifeng Lin, Zhiwei Liu, Longgang Wang

**Affiliations:** 1 Key Laboratory of Applied Chemistry, Hebei Key Laboratory of Heavy Metal Deep-Remediation in Water and Resource Reuse, College of Environmental and Chemical Engineering, Yanshan University, Qinhuangdao 066004, China; lx0406x@163.com (X.L.); hy1201024@126.com (Y.H.); 19991641458@163.com (J.Z.); 15032376798@163.com (J.Z.); zwliu@ysu.edu.cn (Z.L.); 2 Department of Molecular Chemistry and Materials Science, Weizmann Institute of Science, Rehovot 76100, Israel; lin.weifeng@weizmann.ac.il

**Keywords:** dopamine, detection, platinum, colorimetric, *Ganoderma lucidum* polysaccharide

## Abstract

The sensitive and selective detection of dopamine (DA) is very important for the early diagnosis of DA-related diseases. In this study, we reported the colorimetric detection of DA using *Ganoderma lucidum* polysaccharide (GLP) stabilized platinum nanoclusters (Pt_n_-GLP NCs). When Pt_600_-GLP NCs was added, 3,3’,5,5’-tetramethylbenzidine (TMB) was rapidly catalyzed and oxidized to blue oxTMB, indicating the peroxidase-like activity of Pt_600_-GLP NCs. The catalytic reaction on the substrate TMB followed the Michaelis-Menton kinetics with the ping-pong mechanism. The mechanism of the colorimetric reaction was mainly due to the formation of hydroxyl radical (•OH). Furthermore, the catalytic reaction of Pt_600_-GLP NCs was used in the colorimetric detection of DA. The linear range for DA was 1–100 μM and the detection limit was 0.66 μM. The sensitive detection of DA using Pt-GLP NCs with peroxidase-like activity offers a simple and practical method that may have great potential applications in the biotechnology field.

## 1. Introduction

As we all know, dopamine (DA) is an important catecholamine neurotransmitter in the nervous system, which plays a key role in human metabolism. Abnormal levels of dopamine in body fluids can cause various neurological diseases, such as schizophrenia or Parkinson’s disease [1]. Moreover, it has profound significance to control DA at a level suitable for the normal activities of human cells [2]. Therefore, accurate and convenient detection of DA is an important and valuable work in the field of biology. To date, many different methods have been developed to detect DA, including fluorescence [3], electrochemistry [4], and colorimetric methods [5]. It is worth noting that colorimetric methods have always attracted attention due to its simple operation and visual observation. In recent years, some substances with peroxidase-like properties have been considered to be essential in colorimetric sensor platforms. It overcomes the shortcomings of natural horseradish peroxidase (HRP), such as high cost, poor stability, difficult preparation, and storage. Therefore, this kind of artificial peroxidase has been widely studied.

A variety of substances with peroxidase-like properties sprung up like mushrooms, from noble metal nanoparticles [6], metal oxides [7], to multi-metal compounds [8]. This peroxidase-like activity of nanozymes has been used in colorimetry to detect many biologically related substances, such as H_2_O_2_ [9], glucose [10], DA [11], and glutathione [12]. While great progress has been made, there are still some difficulties and challenges in the study of nanozymes. Compared with other artificial enzymes, they have poor stability, low activity, and biocompatibility. Therefore, it is very much expected to explore chemically stable and environmentally friendly nanozymes as simulants in bioassays and disease diagnosis.

Platinum is an important element in chemical industries. Nanozymes for platinum have been extensively studied because of their enzyme-like activities [13], but their preparation still faces challenges. The preparation usually uses some harmful materials, such as carcinogenic polymer materials [14] and flammable chemical reducing agent NaBH_4_ [15]. In addition, there are problems in controlling the size and structure of nanozymes [16,17]. Many reported Pt nanomaterials have relatively large particle sizes, which means a small specific surface area and relatively low atom utilization [18,19]. They are thermodynamically unbalanced and tend to aggregate during chemical reactions, resulting in a loss of activity. Consequently, controlling the shape, size and dispersion of nanoparticles is the key factor in achieving high stability and activity. Recently, polysaccharide as a green biomaterial has attracted people’s attention, which is widely used as a carrier material due to its good biocompatibility [10]. *Ganoderma lucidum* polysaccharide (GLP) is the active component extracted from *Ganoderma lucidum*, and it is a natural biological macromolecule. It has beneficial physiological functions in immune regulation, anti-tumor, anti-oxidation, hypoglycemia, and regulation of intestinal health, so it has received extensive attention [20,21,22]. The biological activity of GLP is correlated with its composition, three-dimensional structure, and helical conformation [20]. GLP contains β-(1→6)-, β-(1→4)-, and β-(1→3)-D-glucopyranosyl residues in the backbone and sidechains [23]. Moreover, there are many active groups in the structure of GLP, such as hydroxyl and carboxyl, so it can be easily chemically and biochemically modified [20,24]. GLP has high biocompatibility, so it is an ideal material for green preparation of platinum-based nanozymes.

**Scheme 1:**
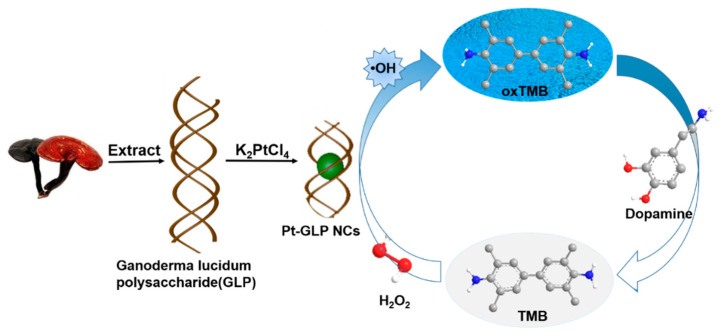
The synthesis of Pt-GLP NCs and the schematic diagram of dopamine detection.

In this work, we prepared GLP-stabilized Pt nanoclusters (Pt_n_-GLP NCs), where GLP was used as a stabilizer and reducing agent. Pt_n_-GLP NCs had a small size for Pt nanoclusters (Pt NCs) and peroxidase-like activity. GLP protected the wrapped nanoparticles from aggregation, resulting in particles with higher stability. To demonstrate the practical application of Pt_n_-GLP NCs, we utilized the peroxidase-like activity of the nanozyme to develop a colorimetric method for the visual and sensitive detection of dopamine (Scheme 1). This method has the potential for the development of bio-related detection of actual samples.

## 2. Results and Discussion

### 2.1. Structure Characterizations

**Figure 1:**
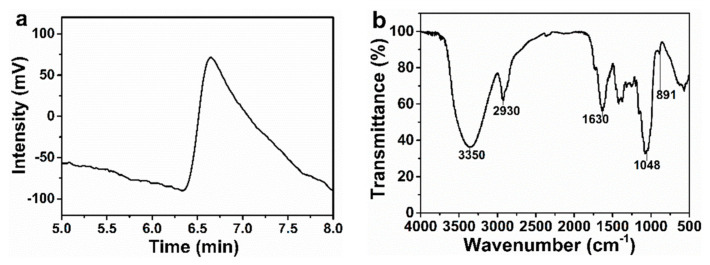
(**a**) GPC determination and (**b**) infrared spectrum of GLP.

Natural plant polysaccharides are formed by various monosaccharides with glycosidic linkages. Plant polysaccharides also have high molecular weight and various biological activities. GLP is a functional substance obtained from *Ganoderma* and a potent clinical agent [25]. We used GPC to determine the relative molecular weight of polysaccharides, and the results are shown in Figure 1a. The M_w_ of GLP was 1.77 × 10^5^ g mol^−1^, and the M_n_ was 1.70 × 10^5^ g mol^−1^. The polydispersity coefficient of GLP was calculated by M_w_/M_n_ = 1.0412, indicating that GLP was a narrowly distributed polydisperse system [26].

The FTIR spectrum of GLP was further measured. Due to the hydroxyl stretching vibration, Figure 1b shows a broad stretching peak at 3350 cm^−1^, while for the C–H stretching vibration, it shows weak absorption at 2930 cm^−1^. The absorbance at 1630 cm^−1^ indicated the presence of a carbonyl group. In addition, the main absorption of C–O stretching (1048 cm^−1^) indicated that the structure of the sugar was pyranose configuration. Moreover, the band at 891 cm^−1^ was the characteristic absorption of β-linkage of pyranose [27].

**Figure 2:**
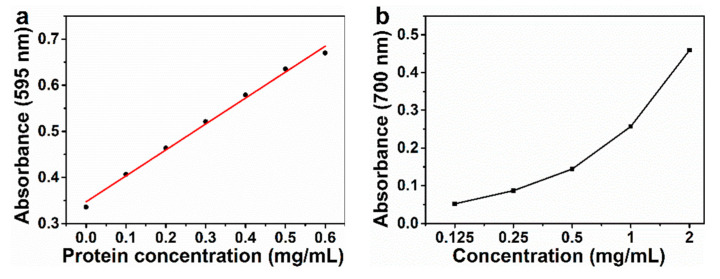
(**a**) The protein content standard curve at 595 nm and (**b**) the reducing ability curve of GLP at 700 nm.

As shown in Figure 2a, the standard curve of protein content was measured by the Coomassie Brilliant Blue method. The equation was *y* = 0.561 *x* + 0.347. The result showed that the purity of the polysaccharide was about 92%. GLP can reduce potassium ferricyanide to potassium ferrocyanide, and ferrous ions can react with ferric chloride to produce Prussian blue, which has a maximum absorption peak at 700 nm. Thus, the reducing ability of GLP was determined by this method. As shown in Figure 2b, the absorbance at 700 nm increased with the increasing concentration of GLP. When the concentration of GLP reached 2 mg/mL, the corresponding absorbance reached 0.459. This indicated that the reducing ability of GLP increased with the increase of concentration of GLP. The reducing ability of GLP was attributed to a large number of hydroxyl and aldehyde groups of GLP, which is a chain compound with a triple-helical structure [20].

**Figure 3:**
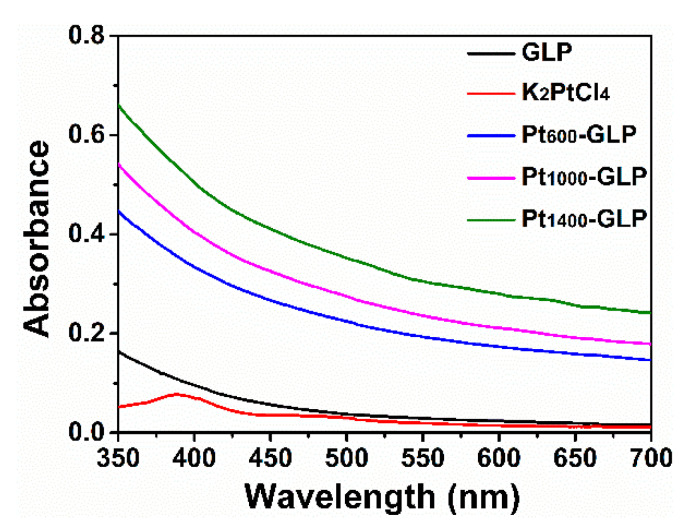
UV-Vis spectra of GLP, K_2_PtCl_4_, and Pt_n_-GLP NCs (n = 600, 1000, and 1400).

The high purity and reducing ability of GLP were beneficial to the generation of noble metal nanomaterials [20]. In this study, Pt NCs were prepared by incubating GLP with K_2_PtCl_4_. As shown in Figure 3, K_2_PtCl_4_ showed a characteristic absorption peak at 390 nm. After K_2_PtCl_4_ and GLP were incubated for 12 h, the absorption peak of K_2_PtCl_4_ solution at 390 nm disappeared, indicating that Pt^2+^ was reduced to Pt^0^ by GLP. Compared with the use of traditional chemical reducing agents such as NaBH_4_, the use of GLP was greener and the reaction was milder.

**Figure 4:**
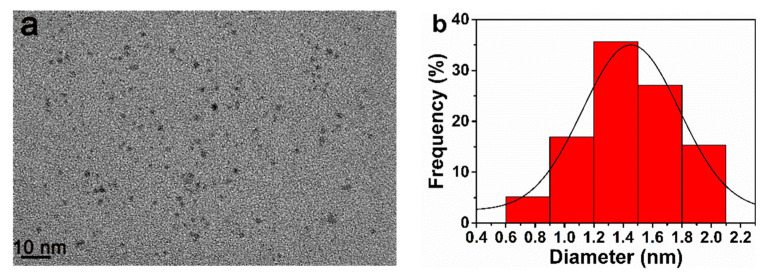
(**a**) HRTEM image and (**b**) histogram of Pt_600_-GLP NCs.

Metal nanoparticles exhibit unique physical and chemical properties due to their small size and large specific surface area. In general, smaller particles show better catalytic efficiency, because smaller nanoparticles possess a larger surface-to-volume ratio [28]. Here, the size of Pt NCs inside Pt_600_-GLP NCs was measured by HRTEM. As shown in Figure 4, the Pt NCs inside Pt_600_-GLP NCs existed in a good monodisperse state, and the calculated average diameter of Pt NCs inside Pt_600_-GLP NCs was 1.44 ± 0.34 nm. Supplementary Materials Figure S1 showed that the Pt NCs inside Pt_600_-GLP NCs and Pt_1000_-GLP NCs had a good dispersion state, respectively. The size of Pt NCs inside Pt_1000_-GLP NCs and Pt_1400_-GLP NCs were 2.12 ± 0.72 nm and 2.34 ± 0.54 nm, respectively. These results indicated that Pt NCs inside Pt_n_-GLP NCs had a small size within 1–3 nm and narrow size distribution, which may be contributed by the mild reducing ability of GLP [29]. The specific surface area of the nanoparticles was calculated by the surface area and volume method, and the specific surface area was 4.41 ± 1.46 nm^2^/nm^3^ for Pt_600_-GLP NCs, 3.20 ± 1.54 nm^2^/nm^3^ for Pt_1000_-GLP NCs, and 2.59 ± 0.71 nm^2^/nm^3^ for Pt_1400_-GLP NCs. Thus, Pt_600_-GLP NCs had the largest specific surface area, and they were used for subsequent experiments.

**Figure 5:**
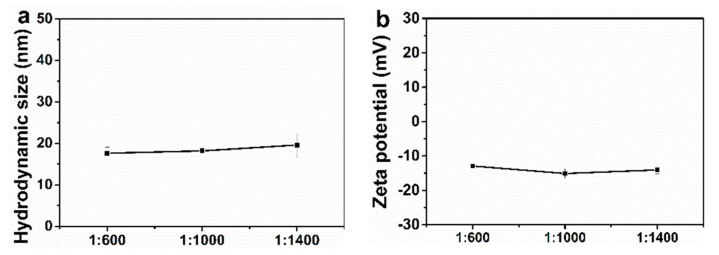
(**a**) The hydrodynamic size and (**b**) zeta potential of Pt_n_-GLP NCs in aqueous solution.

The hydrodynamic size and zeta potential of Pt_n_-GLP NCs were measured using DLS technology [30]. As shown in Figure 5a, the hydrodynamic size was 17.63 ± 1.45 nm for Pt_600_-GLP NCs, 18.18 ± 0.03 nm for Pt_1000_-GLP NCs, and 19.61 ± 2.87 nm for Pt_1400_-GLP NCs, respectively. The hydrodynamic size of Pt_n_-GLP NCs included the GLP molecule, which was much bigger than the size of Pt NCs as measured using TEM. Furthermore, the zeta potential of Pt_n_-GLP NCs was also measured in water. GLP is a chain compound with a triple-helical structure. Figure 5b shows that the zeta potential was −12.93 ± 2.59 mV for Pt_600_-GLP NCs, −15.1 ± 1.37 mV for Pt_1000_-GLP NCs, and −14.08 ± 1.15 mV for Pt_1400_-GLP NCs, respectively. Thus, Pt_n_-GLP NCs had a hydrodynamic size from 17.6 to 19.6 nm and a slightly negative charge [29].

### 2.2. Peroxidase-Like Activity

**Figure 6:**
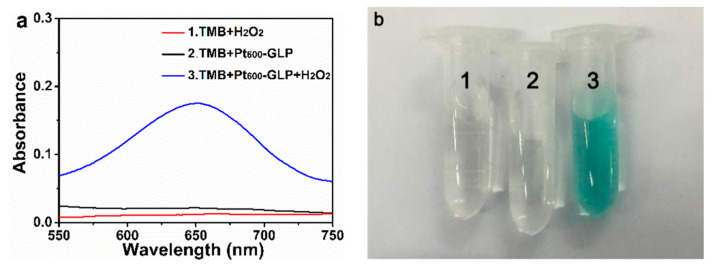
(**a**) UV-Vis spectra and (**b**) picture of the reaction product: (1) TMB + H_2_O_2_, (2) TMB + Pt_600_-GLP NCs, and (3) TMB + Pt_600_-GLP NCs + H_2_O_2_. Concentrations: TMB, 0.6 mM; Pt, 0.78 mM; H_2_O_2_, 0.3 M.

The peroxidase-like activity of Pt_600_-GLP NCs was studied using TMB as the substrate in the presence of H_2_O_2_. As shown in Figure 6a, the TMB + H_2_O_2_ + Pt_600_-GLP NCs group had the maximum absorption peak at 652 nm, and the sample of this group corresponded to a clear blue solution (Figure 6b). Conversely, in the absence of H_2_O_2_ or Pt_600_-GLP NCs, the color of the solution did not change. Therefore, these results demonstrated that Pt_n_-GLP NCs had peroxidase-like activity. Other noble metal nanoparticles, such as platinum nanoparticles [10] and rhodium nanoparticles [31], have also been reported, exhibiting peroxidase-like activity.

**Figure 7:**
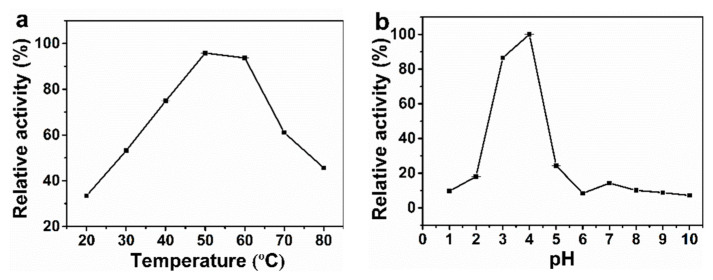
Effects of (**a**) temperature and (**b**) pH on the peroxidase-like property of Pt_600_-GLP NCs.

To optimize the reaction conditions, the effects of temperature and pH on the catalytic activity were further investigated. As shown in Figure 7a, the relative catalytic activity of Pt_600_-GLP NCs reached the highest when the temperature was 50 °C, which was optimal for the reaction. The relative activity of Pt_600_-GLP NCs between 30 and 70 °C was above 50%. Similarly, Figure 7b showed that the optimal pH for Pt_600_-GLP NCs was 4.0. The optimal pH was consistent with the optimal activity of nanozymes reported by Wu et al. [32]. The optimum temperature and pH were correlated with the activity of H_2_O_2_. The optimal conditions were temperature 50 °C and pH 4.0, which were used for subsequent experiments.

### 2.3. Steady-State Kinetic

**Figure 8:**
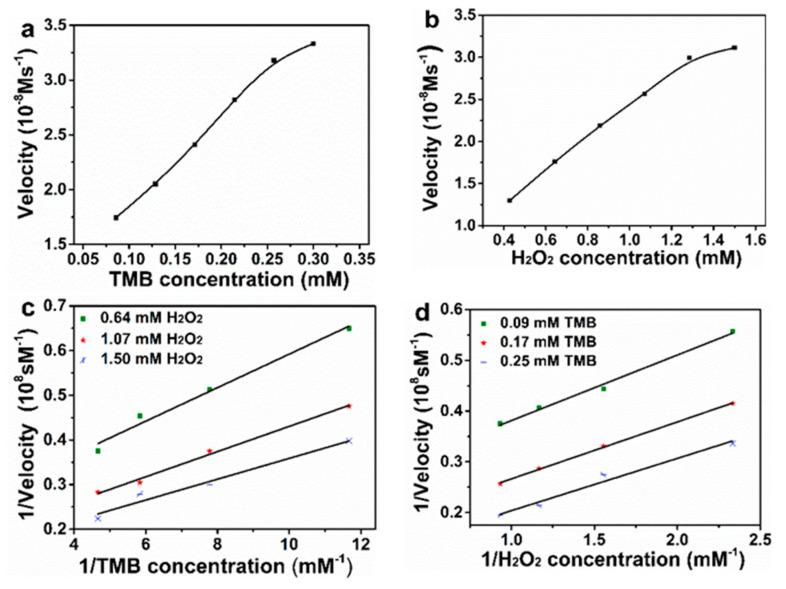
Steady-state kinetic assay and catalytic mechanism of Pt_600_-GLP NCs: (**a**) The H_2_O_2_ was 1.07 mM with varying TMB concentration. (**b**) The TMB was 0.17 mM with varying H_2_O_2_ concentration. (**c**,**d**) Double reciprocal plots of activity of Pt_600_-GLP NCs with the concentration of one substrate (H_2_O_2_ or TMB) fixed and the other varied.

The catalytic activity of Pt_600_-GLP NCs was investigated based on enzyme kinetics theory methods with H_2_O_2_ and TMB as substrates. A range of kinetic data was determined by changing the concentration of H_2_O_2_ or TMB. Figure 8a,b are consistent with the Michaelis-Menten equation for TMB and H_2_O_2_, respectively. The apparent steady-state kinetic parameters *V_max_* and *K_m_* were obtained using the Lineweaver-Burk plots, as shown in Table 1. The *K_mH2O2_* (2.06 mM) and *K_mTMB_* (0.17 mM) of Pt_600_-GLP NCs with substrates were both lower than the *K_m_* value corresponding to HRP. These data indicated a greater affinity between Pt_600_-GLP NCs and the substrate. As shown in Figure 8c,d, several parallel ramp data lines conformed to the characteristics of a ping-pong mechanism. The kinetic mechanism of Pt_600_-GLP NCs was in accordance with the enzyme-catalyzed ping-pong mechanism.

### 2.4. Catalytic Mechanism

**Figure 9:**
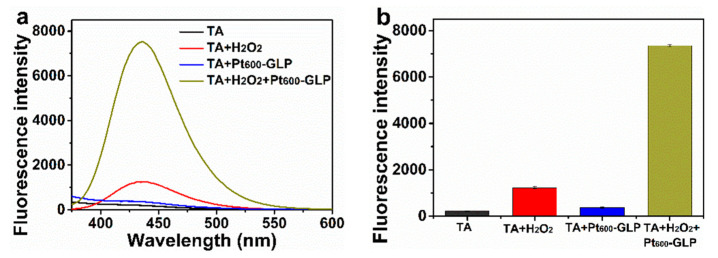
(**a**) Fluorescence spectra and (**b**) histograms of fluorescence intensity of TA + H_2_O_2_ + Pt_600_-GLP NCs group and control groups after 12 h. The final concentrations of TA, H_2_O_2_, and Pt were 0.3 mM, 20 mM, and 49 μM, respectively.

The enzymatic reaction mechanism may involve the decomposition of H_2_O_2_, which will generate •OH radicals in the reaction system. To confirm this hypothesis, the formation of •OH radicals was evaluated by using terephthalic acid (TA) as a fluorescent probe, where the TA easily reacted with •OH to form highly fluorescent 2-hydroxyterephthalic acid. As shown in Figure 9, the system TA + H_2_O_2_ + Pt_600_-GLP NCs generated the highest peak at 435 nm and the largest amount of •OH. The fluorescence intensity of the system TA + H_2_O_2_ + Pt_600_-GLP NCs reached 7351. Pt_600_-GLP cannot effectively promote TA oxidation in the absence of H_2_O_2_. In addition, the TA + H_2_O_2_ system produced a small amount of •OH and its corresponding fluorescence intensity was 1227. Other systems were much lower than that of the TA + H_2_O_2_ + Pt_600_-GLP NCs system. The high fluorescence intensity indicated that the peroxidase-like activity of Pt_600_-GLP NCs was achieved by •OH radical mechanism. The results were similar to the previous results of Ivanova et al. [11]. The intrinsic peroxidase activity of Pt-GLP NCs was mainly derived from Pt NCs [34], rather than the GLP. Thus, these results confirm the generation of •OH radicals catalyzed by Pt_600_-GLP NCs, and •OH radicals were the main reactive species in the peroxidase-like enzymatic reaction.

### 2.5. Detection of Dopamine

**Figure 10:**
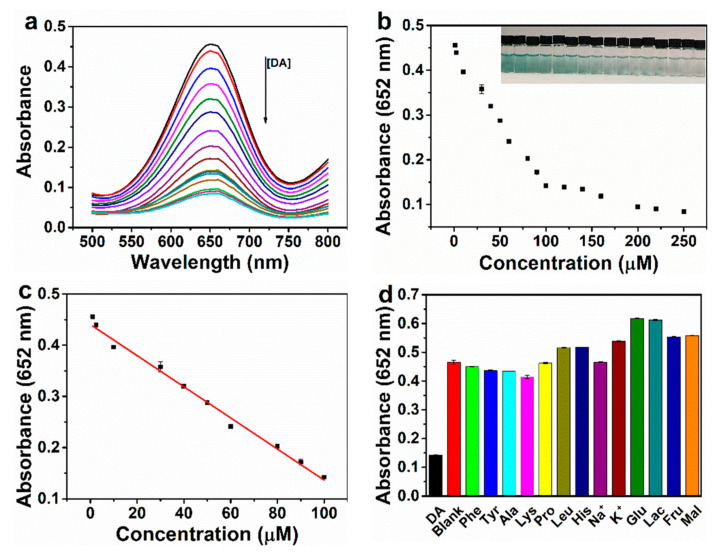
(**a**) The absorption spectra and (**b**) absorbance at 652 nm of mixed solutions when Pt_600_-GLP NCs and TMB systems were added by various concentrations of DA (1–250 μM), (**c**) linear calibration chart of DA detection, and (**d**) the absorbance of dopamine (100 μM) and other interfering substances (1200 μM).

Dopamine (DA) is involved in many neural processes, and the concentration of dopamine is an important signal indirectly reflecting the health status of organisms. We applied a colorimetric method to detect dopamine based on the peroxidase-like reaction of Pt_600_-GLP NCs. Since the DA molecule has certain reducibility, it will cause the blue solution of Pt_600_-GLP NCs to fade after the DA molecule is added into the reaction system Pt_600_-GLP NCs + H_2_O_2_ + TMB [35]. Figure 10a showed the changes of the reaction system solution with a DA concentration of 1 to 250 μM in the UV-Vis spectra. As shown in Figure 10b, the absorbance decreased with the increase of DA concentration, indicating that the blue oxTMB gradually became colorless TMB. Figure 10c showed that A_652nm_ decreased linearly with DA concentration, and the linear response range of DA concentration was 1 to 100 μM. The equation of the standard curve of DA detection was A = −0.00304 C_DA_ + 0.44049 (R^2^ = 0.991). In addition, the limit of detection (LOD) of DA was as low as 0.66 μM. Compared with other nanoparticles previously reported (Table 2), Pt_600_-GLP had a wide linear concentration and low detection limit. Therefore, Pt-GLP NCs had higher sensitivity than other mimic enzymes in the detection of DA.

In addition, the selectivity of this method was evaluated. From Figure 10d, it could be clearly observed that the absorbance of the Pt_600_-GLP NCs + TMB + H_2_O_2_ system was reduced remarkably with the addition of DA. Thus, the system exhibited better selectivity in the detection of DA (100 μM) than other interfering substances (1200 μM). The interfering substances included phenylalanine, tyrosine, alanine, lysine, proline, leucine, histidine, Na^+^, K^+^, glucose, lactose, fructose, and maltose.

In addition, a standard addition method was used for recovery experiments, in which DA samples with different concentrations were spiked into human serum samples for analysis. As shown in Table 3, the acceptable recovery rate ranged from 96.66% to 98.80%, and the relative standard deviation was less than 4%. Therefore, this method had a good performance in detecting DA in real samples.

## 3. Materials and Methods

### 3.1. Chemicals and Materials

*Ganoderma lucidum* was purchased from the local drugstore (Guangyuan, Qinhuangdao in China). Hydrochloric acid (HCl), acetic acid (HAc), sodium acetate (NaAc), terephthalic acid (TA), sodium hydroxide (NaOH), potassium tetrachloroplatinate (II) (K_2_PtCl_4_), 3,3′,5,5′-tetramethylbenzidine (TMB), hydrogen peroxide (H_2_O_2_, 30%), and dopamine hydrochloride (DA) were purchased from Aladdin (Shanghai, China).

### 3.2. Extraction of Polysaccharide

GLP was extracted using the hydrothermal extraction method reported by a previously reported study [42]. The dried *Ganoderma lucidum* powder was mixed with deionized water at a ratio of 1:50 for water to the powder. The powder was extracted in a 60 °C constant temperature water bath for 3 h. After extraction, the extract was centrifuged at 4500 rpm for 10 min. Secondly, the supernatant was incubated in a 50 °C water bath and decolorized with 30% hydrogen peroxide for 2 h. Then, the extract was deproteinized by the Savage method, and the organic reagents were removed by rotary evaporation. Finally, it was dialyzed with deionized water for 48 h to obtain GLP after freeze-drying.

### 3.3. Determination of Molecular Weight

The relative molecular weight of polysaccharides was obtained by Shimadzu gel permeation chromatography (GPC, Shimadzu, Kyoto, Japan). The mobile phase was water, the flow rate was 1 mL/min, the injection volume was 20 μL, the column temperature was 30 °C, and the chromatographic curve of 1 mg/mL polysaccharide solution was recorded.

### 3.4. Determination of Reducing Power

One milliliter (1 mL) polysaccharide solutions with different concentrations were put into a 10 mL PE tube respectively, and 2.5 mL phosphate buffer solution at pH = 6.6 was added into the PE tube and mixed well. Then, 2.5 mL 1% potassium ferricyanide solution was added. The PE tube was placed in a 50 °C water bath for 20 min, then removed and quickly cooled, and 2.5 mL of 10% trichloroacetic acid was added to the cooled solution and centrifuged at 3000 r/min for 10 min. In another PE tube, 2.5 mL of supernatant was added, then 0.5 mL of 0.1% ferric chloride and 2.5 mL of distilled water were also added and mixed well. The absorbance of the solution at 700 nm was determined by a UV-Vis spectrophotometer (Beijing Purkinje General Instrument Co., Ltd, Beijing, China). In the blank control group, deionized water was used instead of polysaccharide solution, and the above steps were repeated. The reducing power was calculated by sample absorbance minus control absorbance.

### 3.5. Purity Determination

The protein content in the extracted polysaccharide was obtained by the Coomassie Brilliant Blue G-250 (Aladdin, Shanghai, China) staining method. In short, 1 mg/mL protein solution was prepared as the standard solution and then diluted into different concentrations. The Coomassie brilliant blue G-250 reagent was then added to each solution and thoroughly mixed. After standing for 5 min, the absorbance of the solution at 595 nm was measured by using a UV-Vis spectrophotometer. Thus, the standard curve between absorbance and protein concentration was determined. The absorbance of extracted polysaccharide solution was measured at 595 nm, and the protein content was calculated according to the standard curve.

### 3.6. Synthesis and Characterizations of Pt_n_-GLP NCs

GLP solution (1 mg/mL) was incubated with K_2_PtCl_4_ (1 mM) at 60 °C for 12 h. The molar ratios of K_2_PtCl_4_ and GLP solution were 600:1, 1000:1, and 1400:1, respectively. Then, the reaction solution was dialyzed in deionized water for 24 h. Different ratios of Pt_n_-GLP NCs (n = 600, 1000, and 1400) were obtained.

All the UV-Vis measurements were carried out using a UV-TU1810PC spectrophotometer (Beijing Purkinje General Instrument Co., Ltd, Beijing, China). The incubation equipment was a Thermo Shaker (MSC-100) (Hangzhou, China). The size of Pt NCs was evaluated by transmission electron microscopy (TEM, JEM-1230EX, Hitachi, Tokyo, Japan). Dynamic light scattering (DLS, Malvern, Worcestershire, UK) was used to determine the hydrodynamic size and zeta potential of samples.

### 3.7. Measurements for the Peroxidase-Like Catalytic Activity

To perform the catalytic reaction, 50 μL Pt_600_-GLP NCs (C_Pt_ = 0.78 mM) were incubated at 25 °C for 3 min in a 2 mL PE tube containing 400 μL HAc-NaAc buffer (0.2 M, pH 4.0), and 300 μL TMB (0.6 mM) as substrate was added for 3 min. Then, 100 μL H_2_O_2_ (0.3 M) was added. After reacting for 5 min, the absorption spectra of the mixture were measured. All TMB was dissolved in 0.2 M HAc-NaAc buffer solution.

The catalytic activity of Pt_600_-GLP NCs under different pH (1.0–10.0) was evaluated as follows: 100 μL of Pt_600_-GLP NCs (0.78 mM) and 300 μL of HAc-NaAc buffer (pH 1.0–10.0, 0.2 M) were incubated for 3 min, 900 μL of TMB (0.6 mM) was mixed for 3 min, then 100 μL of H_2_O_2_ (0.3 M) was added for 5 min. The absorbance was recorded at 652 nm. Furthermore, the effect of temperature on the catalytic activity was studied by a similar method. Different reaction temperature from 20 to 80 °C was used to select the most suitable reaction temperature.

### 3.8. Kinetic Analysis

The kinetic measurement of Pt_600_-GLP NCs was carried out in time scanning mode under optimal conditions by monitoring the absorbance change at 652 nm on a UV-Vis spectrophotometer. First, 100 μL Pt_600_-GLP NCs (C_Pt_ = 0.78 mM) were kept in different volumes of HAc-NaAc buffer solution. Then, a constant 900 μL TMB (0.6 mM) or 100 μL H_2_O_2_ (0.3 M) was added as a substrate and reacted for 5 min. Finally, after adding different volumes of H_2_O_2_ (0.3 M) or TMB (0.6 mM) solution, the absorbance changes over time were recorded. The Michaelis-Menten constant (*K_m_*) was calculated by the Lineweaver-Burk plot:

(1) $$\frac{1}{v}=\frac{K_{m}}{V_{max}}\frac{1}{\left[S\right]}+\frac{1}{V_{max}}$$

where *v*, *K_m_*, *V_max_*, and [*S*] represent the initial velocity, Michaelis constant, the maximal reaction velocity, and the concentration of the substrate, respectively.

### 3.9. Mechanism Detection

To study the peroxidase-like enzymatic mechanism of Pt_600_-GLP NCs, terephthalic acid (TA) was used as a fluorescent probe (Hitachi, Tokyo, Japan) to specifically capture the hydroxyl radicals (•OH) possibly generated. Typically, in the PE tube, 900 μL of terephthalic acid (0.5 mM) solution and 500 μL of sodium acetate buffer (0.2 M, pH 4.0) were added. Next, the reaction was carried out in a Thermo Shaker at 40 °C for 3 min. Then, 100 μL Pt_600_-GLP NCs (C_Pt_ = 0.78 mM) was added and the reaction continued for 3 min. After adding 100 μL H_2_O_2_ (0.3 M) for 12 h, the final fluorescence of the solution was measured. The final concentrations of TA, H_2_O_2_, and Pt were 0.3 mM, 20 mM, and 49 μM, respectively.

### 3.10. Dopamine Detection

For DA detection, Pt_600_-GLP NCs (100 μL, C_Pt_ = 0.78 mM) was added into a solution containing TMB (0.6 mM) and H_2_O_2_ (6 mM). After 5 min, different amounts of DA were added to the solution. After another 5 min, the absorbance at 652 nm was recorded by spectroscopic measurements.

For evaluating the selectivity of the colorimetric method, some typical interfering agents such as phenylalanine, tyrosine, alanine, lysine, proline, leucine, histidine, Na^+^, K^+^, glucose, lactose, fructose, maltose (1200 μM), and dopamine (100 μM) were further investigated.

The effectiveness of dopamine was tested by measuring the recovery. First, the serum was collected by centrifugation. Secondly, the 1 μL serum solution was mixed with DA of different concentrations (15, 30, 45 μM) to obtain the sample solution to be tested. Finally, the absorbance at 652 nm was recorded by the established method.

## 4. Conclusions

In summary, GLP was used as a reducing agent and stabilizer to synthesize platinum nanoclusters. The size of Pt NCs inside Pt_n_-GLP NCs ranged from 1.10 to 2.88 nm. Pt_n_-GLP NCs had the peroxidase-like catalytic activity. The best conditions for catalytic activity were pH 4.0 and temperature 50 °C. The catalytic kinetic process of Pt_600_-GLP NCs conformed to the typical Michaelis-Menten equation, and their affinity for both substrates was bigger than that of HRP. Their catalytic process conformed to the ping-pong mechanism. Using the peroxidase activity exhibited by Pt_n_-GLP NCs, a colorimetric method for dopamine detection was established. The linear range of the detection method was 1–100 μM, and the detection limit was 0.66 μM. Therefore, the DA concentration can be quantitatively detected by the colorimetric method, which had advantages of a wide detection range, low detection limit, and high selectivity of DA. This method provides a new application of Pt_n_-GLP NCs in bio-related detection.

## Figures and Tables

**Table 1:** Comparison of the kinetic parameters of Pt_600_-GLP NCs and HRP.

Catalyst	Substrate	*K_m_* (mM)	*V_max_* (10^−8^ M s^−1^)	Reference
Pt_600_-GLP NCs	TMB	0.17	5.04	this work
Pt_600_-GLP NCs	H_2_O_2_	2.06	7.51	
HRP	TMB	0.434	10.00	[33]
HRP	H_2_O_2_	3.70	8.71	

**Table 2:** Comparison of the detection of dopamine using Pt_600_-GLP NCs with other reported nanozymes.

Materials	Linear Range (μM)	Detection Limit (μM)	Method	Reference
Au-Cu_2_O/rGO	10–90	3.9	Electrochemistry	[36]
Lac-SBA15	4–500	4.05	Electrochemistry	[37]
GCE/N-rGO-Au	3–100	2.4	Electrochemistry	[38]
WS_2_ QDs	3–50	3.3	Fluorescence	[39]
ACP-CDs	3–20	1	Fluorescence	[40]
Pt/hBNNSs-5	2–55	0.76	Colorimetry	[11]
Pt/CoSn(OH)_6_	5–60	0.76	Colorimetry	[41]
Pt_600_-GLP NCs	1–100	0.66	Colorimetry	this work

**Table 3:** Results for the determination of DA in human serum samples (n = 3) ^1^.

Sample	Added Amount (μM)	Found Amount (μM)	Recovery (%)	RSD (%)
Serum	15	14.85	99.03	2.11
Serum	30	28.99	96.66	1.28
Serum	45	44.46	98.80	3.23

^1^ Dopamine is widely present in the nervous system, and the normal level of DA in serum is between 0.01 and 1 μM. Since the concentration of DA in the real sample is very low and the amount spiked in the system is very small, the concentration of DA in the initial sample can be considered as zero.

## Data Availability

The data presented in this study are available on request from the corresponding author.

## Supplementary Materials

The following are available online, Figure S1: TEM image and histogram of Pt_1000_-GLP NCs and Pt_1400_-GLP NCs.
