# Supplementary material for: Peroxidase-Like Platinum Clusters Synthesized by Ganoderma lucidum Polysaccharide for Sensitively Colorimetric Detection of Dopamine
Source: Molecules. 2021 May 6;26(9):2738. doi: 10.3390/molecules26092738 (PMC8125108; doi:10.3390/molecules26092738)
Supplement: Supplementary file 1 [file molecules-26-02738-s001.zip › molecules-1201872-supplementary.pdf]

supplementary materials

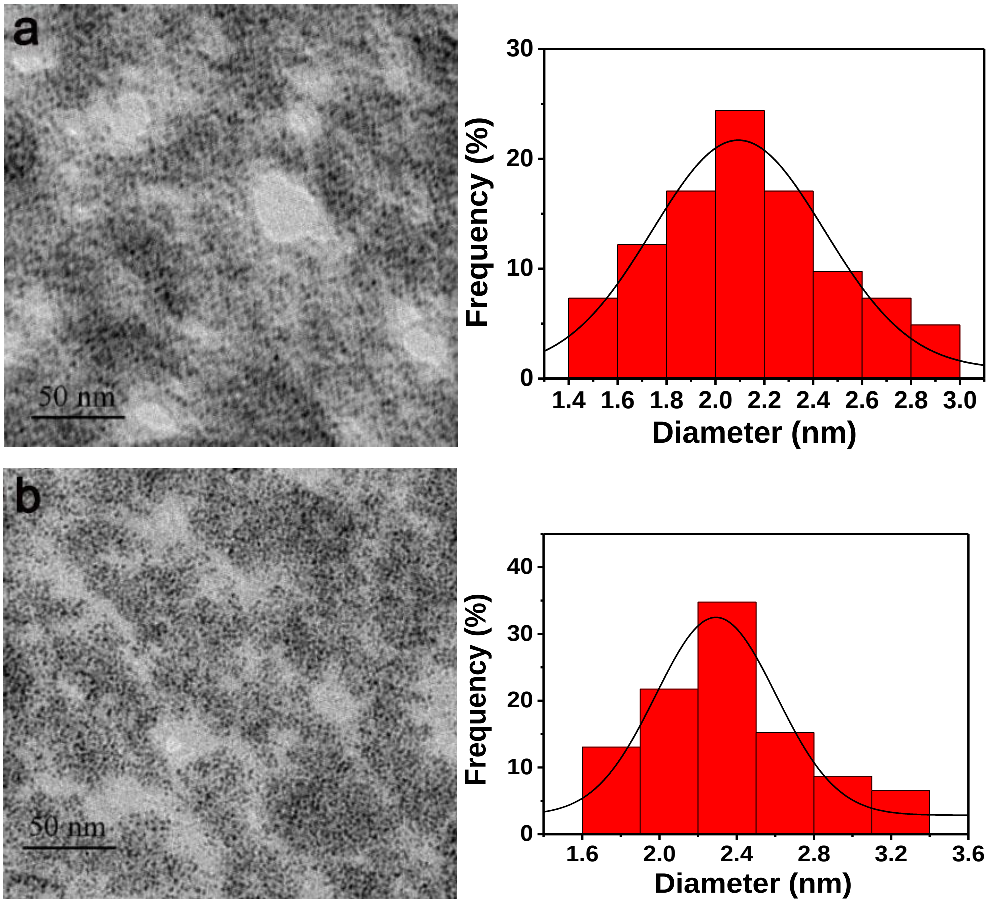

Figure. S1 TEM image and histogram of (a) Pt_{1000}-GLP NCs and (b) Pt_{1400}-GLP NCs.
